# COVID-19 and Pro-environmental Behaviour at Destinations Amongst International Travellers

**DOI:** 10.3389/fpsyg.2022.879300

**Published:** 2022-04-14

**Authors:** Gary Calder, Aleksandar Radic, Hyungseo Bobby Ryu, Antonio Ariza-Montes, Heesup Han

**Affiliations:** ^1^Independent Researcher, Dundee, United Kingdom; ^2^Independent Researcher, Dubrovnik, Croatia; ^3^Department of Food Franchise, College of Health Sciences, Kyungnam University, Changwon, South Korea; ^4^Social Matters Research Group, Universidad Loyola Andalucía, Córdoba, Spain; ^5^College of Hospitality and Tourism Management, Sejong University, Seoul, South Korea

**Keywords:** health belief model (HBM), theory of planned behaviour (TPB), COVID-19, international travellers, pro-environmental behaviour

## Abstract

This paper investigates the COVID-19 pandemic, and its impact on pro-environmental behaviour of individuals travelling internationally for leisure and recreational purposes. The aim of this manuscript is to investigate a conceptual framework created through the examination of current existing literature in the field of tourism science. The conceptual framework, consisting of certain constructs of the health belief model (HBM), and the theory of planned behaviour (TPB), is applied and tested using a partial least-squares-structural equation modelling (PLS-SEM). Data were collected from participants who have travelled internationally before and during the outbreak of the COVID-19 pandemic, and those who plan to travel post-COVID-19 pandemic. Results revealed that the conceptual framework tested positively against existing theory, highlighting the key influencing factors in which COVID-19 is likely to have on future pro-environmental behaviour of individuals travelling internationally for leisure and recreational purposes. Moreover, perceived safety threat and outcome expectations have a positive impact on attitude; attitude has a positive impact on behavioural intentions; subjective norm has a positive impact on behavioural intentions, and perceived behavioural control has a positive impact on behavioural intentions. The study results identify practical and theoretical implications for global and travel companies and organisations, presenting opportunities to adjust environmental policies and procedures accordingly, whilst identifying the most effective marketing and management strategies to rebuild a collapsed global travel industry.

## Introduction

This study sets out to investigate the effect of the Coronavirus (COVID-19) pandemic on pro-environmental behaviour amongst individuals travelling internationally for leisure and recreational purposes. Since 2019, the COVID-19 pandemic has brought devastation across the globe, causing turmoil and socioeconomic distress, particularly within the travel and tourism industry (Yazir et al., 2020). Despite scientists previously warning worldwide leaders for many years regarding the possibility of a pandemic, the global society was caught off guard by the devastation caused by COVID-19 (Žižek, 2021).

Recent studies suggest there is a link between the COVID-19 pandemic and environmentally reckless capitalist practises such as the accelerated clearing of rainforests for palm oil. Consequently, this practice destroys the insulation forests offer from zoonotic diseases and has resulted in infections that are transmissible from animals to humans (Gillespie, 2020). Further, greenhouse gases contribute to the gradual increase of the Earth’s temperature (climate change), generated from the by-product of non-renewable energy sources used for economic activities.

According to Han et al. (2020), due to the COVID-19 pandemic, the global travel and tourism sector has faced its most catastrophic crisis in history, resulting in substantial disruption to the sociological behaviours of individuals travelling internationally, whilst causing serious economic impact across the travel industry (Scarlett, 2021). With extreme uncertainty across the travel and tourism sector, understanding the behavioural intention of individuals travelling internationally will allow industry practitioners and researchers to identify fundamental issues within the global travel industry, and to offer solutions to effectively rebuild a collapsed travel economy. Since 2019, according to the most recent data collected the United Nations Conference on Trade and Development (UNCTAD), it is estimated there has been a 74% decrease in internationally inbound tourist arrivals, equating to approximately 1 billion trips, with an estimated loss of between US$1–2.5 trillion during the COVID-19 pandemic (Vanzetti et al., 2021).

Bamberg and Möser (2007) explain that pro-environmental behaviour derives from pro-social motives, such as social anxiety, the influence of future generations, and ecological community awareness. Societal behaviours are commonly categorised as *norm* theories, or normative beliefs (Ajzen, 1991), which are set rules for behaviours that concern the plausibility of an individual approving or disapproving of an expected, or socially accepted behaviour. The power and influence each *norm* holds depend on the self-esteem of the individual, with *personal norms* suggested to have more influence over *subjective norms* and *societal norms*. Further, research studies that focus on eco-friendly behaviour as self-interested behaviour, namely the Theory of Planned Behaviour (TPB), emphasise that cognitive triggers significantly promote “green” and “eco-friendly” behaviour (Ajzen, 1991). In addition, the TPB has been proven to help clarify travellers’ decision-making processes, providing a better understanding for industry professionals and analysts (Han et al., 2020). Although the TPB has been proven to support behavioural intention, it is noted that empirical data regarding TPB within the context of the effects of the COVID-19 pandemic on pro-environmental behaviour is limited. Therefore, insights that would provide a deeper understanding of international travellers’ choice for selecting safer and more eco-friendly tourist travel methods and destinations, are sparse, particularly during the COVID-19 pandemic.

Over the past year, studies suggest that the COVID-19 vaccination rollout has brought the rate of infections and symptoms under control (Centers for Disease Control and Prevention [CDC], 2021). However, the behaviours of international travellers remain unclear, particularly with the awareness, or lack of, regarding the adverse impact capitalists have on the global environment. Therefore, the following research questions will be addressed: (1) How is the COVID-19 pandemic affecting pro-environmental behaviour of leisure-orientated international travellers? (2) How is the COVID-19 pandemic affecting leisure-orientated international travellers’ perceived safety threat and the outcome expectations? (3) How is the COVID-19 pandemic affecting leisure-orientated international travellers’ attitudes, subjective norms and perceived behavioural control toward pro-environmental behaviour?

This paper will contribute to existing theory by effectively expanding the TPB through certain constructs of the Health Belief Model framework. A focus on key concepts, such as attitudes, subjective norms, perceived behavioural control, perceived safety threat, and the outcome expectations will help identify behavioural intentions with the effect of the COVID-19 pandemic, and its impact on an individual’s pro-environmental behaviour.

As previously mentioned, the tourism sector has faced its largest crisis in history, resulting in substantial disruption of the sociological behaviours of international travellers (Han et al., 2020), including a consequential economic impact across the travel and tourism industry (Scarlett, 2021). Inevitably, the outbreak of the COVID-19 pandemic witnessed global public health officials placing orders restricting international travel and activities associated with the spread of the virus (Ojo et al., 2022), such as population movement and person-to-person contact. Thus, by considering the perceived safety threat and outcome expectations related to the health risk of COVID-19, this study will provide a valuable framework to help companies and organisations within the travel and tourism industry address the international traveller’s pro-environmental expectations by providing theoretical and practical implications.

## Literature Review

### The Health Belief Model

The Health Belief Model (HBM) was developed in the 1950s to help explain why individuals fail to participate in programmes to detect and prevent (Rosenstock, 1974). Since its development in the 1950s, the HBM has expanded and evolved globally, supporting diverse cultures and sociopsychological behaviours (Jones et al., 2014). The HBM, consisting of six constructs, includes *risk susceptibility*, *risk severity, benefits to action*, *barriers to action*, *self-efficacy* and *cues to action* (Jones et al., 2014). It is important to note, the first four constructs, namely *risk severity*, *risk susceptibility*, *benefits to action* and *barriers to action*, were developed initially, creating the foundation of the framework, with *cues to action and self-efficacy* helping to explain, or intervene the anticipated outcome expectancies (LaMorte, 2019). Given the nature of the COVID-19 pandemic and its limited research across the global travel industry thus far, there is a significant emphasis on the initial 2 constructs of the HBM, namely *risk susceptibility* and *risk severity*.

#### Risk Susceptibility (Perceived Safety Threat)

Rosenstock (1974) outlines *perceived safety threat* factors that influence individual health-related action. The three factors include (1) *the existence of sufficient concern to make health issues relevant*, (2) *the belief that an individual and their behaviour creates vulnerability to a specific condition*, and (3) *the belief that following recommended health guidelines will reduce susceptibility to the condition*, *at a subjectively justifiable cost*. According to the Centers for Disease Control and Prevention [CDC] (2020), individuals with the highest *perceived safety threat* from the COVID-19 virus are determined by age (*80 and 95% of COVID-19 deaths occurring in people aged 65*+ *and 45*+ *respectively*), suggesting those aged 44 and younger account for only 5% of deaths caused by COVID-19. Further, according to Clark et al. (2020) an estimated 22% of the global population (1.7 billion people) possess one or more underlying health conditions that increases the risk of severe COVID-19 symptoms if infected. In addition, a potential 4% of the world’s population (349 million people) face hospitalisation if infected (London School of Hygienic and Tropical Medicine, 2021).

#### Risk Severity

Many factors influence the likelihood of contracting COVID-19 (Collins, 2021), including possible virus exposure, and whether there is an acquired immunity from previous infections. COVID-19 is a highly transmissible virus, spreading from human to human through inhalation, or contact with infected droplets (Wu et al., 2021), or direct contact via saliva, coughing or sneezing within a range of approximately 1–1.5 m (Mohapatra et al., 2020). Recent studies have suggested that risk perceptions and subjective norms play a significant role in the decision to cancel or postpone international travel due to the COVID-19 pandemic (Ojo et al., 2022). Further, according to Sorci et al. (2020) COVID-19 case severity and fatality rates varied from country to country as the pandemic continued to spread. Moreover, in a research article conducted by Cartaxo et al. (2021) countries with the highest COVID-19 risk and severity based on the speed of the virus, the incidence of infection and population size, included India, the United States and Brazil, while the countries with the lowest potential risk and severity due to the COVID-19 exposure included New Zealand and Germany. A study conducted by Shadmi et al. (2020) further emphasises concern regarding the inequitable spread of COVID-19 in densely populated areas as well as the limited and poor access to high-quality health and medical care systems, particularly in countries with lower socioeconomic status. Hence, individuals may consider alternate travel destinations, such as New Zealand and Germany, based on a lower risk severity, whilst avoiding countries such as India, the United States, and Brazil, that possess a higher risk severity (Cartaxo et al., 2021). Over the past year, the COVID-19 vaccine rollout has substantially reduced the risk of infection by 91% for those who have received full vaccination (Centers for Disease Control and Prevention [CDC], 2021). However, it is important to note that the COVID-19 vaccine rollout has been uneven across the globe (Tatar et al., 2021).

### Theory of Planned Behaviour

The TPB was created by social psychologist Icek Ajzen as a general model to predict and explain the performance of human behaviour and behavioural intention. Since its development, TPB has tested, progressed, and raised questions in many social science fields, resulting in considerable interest amongst practitioners and researchers in the field (Tornikoski and Maalaoui, 2019). The TPB explains the formation of behavioural intention via three precursors: *behavioural attitudes*, *subjective norms*, and *perceived behavioural control* (Ajzen, 1991). Due to its complexity, explaining human behaviour is a difficult task, and in general terms, TPB is supported significantly by observational and experimentational studies (Ajzen, 1991). Further, conclusive data outlining *behavioural attitudes*, *subjective norms* and *perceived behavioural control* has provided compelling explanations against actual human behaviour and predicted future trends. Recent studies that utilised the three constructs of the TPB framework identified behavioural patterns relating to following COVID-19 socially distancing rules, demonstrating positive results with individuals changing their behavioural attitude, adhering to social pressures whilst recognising their ability to perform the required behavioural outcome. This behavioural change is triggered by the individual’s fear of contracting COVID-19 and the potentially fatal symptoms that COVID-19 carries (Frounfelker et al., 2021). It is suggested that this highlights the importance of extending the TPB model by amalgamating certain constructs of the HBM framework, such as the previously mentioned *risk susceptibility* and *risk severity*, with behaviours portrayed by individuals adhering to social-distancing rules.

#### Attitude

*Attitude* toward human behaviour refers to the individual evaluation or appraisal in question, and whether certain decisions are favourable, or unfavourable (Tornikoski and Maalaoui, 2019). In broad terms, we, as humans, support some policies, whilst undermining others; we make certain decisions and reject others (Crano and Prislin, 2008). The more optimistic an individual’s thought process is toward the aftereffect of a particular decision, the more favourable their attitude is toward engaging in such behaviours, particularly toward the ease of engagement. It is important to note, Azlan et al. (2020) emphasise that an effective outcome depends on the cooperation and compliance of all members of society. During the initial outbreak of the pandemic, Perrotta et al. (2021) conducted a survey, with 71,612 questionnaires collected between 13 March 2020 and 19 April 2020 from participants across the globe. It was found that individuals were wearing face coverings before mandatory rules were put in place by governing officials. Further, Abolfotouh et al. (2021) conducted a study with 2,470 adults in Saudi Arabia between 17 April 2020 and 29 April 2020 and found positive attitudes toward social distancing (97.1%), the government’s response to the crisis (95%), and individual hygiene standards (93.2%), whilst identifying negative attitudes toward COVID-19 deaths (83.7%), the impact of the pandemic globally (81.0%), and those who had tested positive to the virus (77.8%).

#### Subjective Norms

According to Ojo et al. (2022), in addition to the risk of contracting COVID-19 while travelling, an individual’s decision to engage in recreational travel depends on the subjective norms of others, especially with the influence of public appeals from government officials to adhere to social distancing and travel restrictions. *Subjective norms* affect behavioural intentions and are explained by Ajzen (1991) as the perceived pressure from society to conduct, or not to conduct a specific behaviour. In essence, *subjective norms* refer to the persuasion from an individual, or individual’s family member, friend, loved one, work or business partner, toward a particular decision-making process. During the period of the pandemic, government officials and global leaders have suggested the use of COVID-19 passports (Michaels, 2021). A recent study conducted by Cerda and García (2021), identified that 28% of the 370 participants surveyed were hesitant to receive the COVID-19 vaccine for three main reasons: adverse side effects and extent of risk, the lack of vaccine knowledge, and the preference for others to be vaccinated first. This conclusive data is backed by Han et al. (2020), highlighting the significant influence of the perceived psychological risk when individuals consider travelling internationally, particularly whilst considering destinations that are perceived safer than others. Moreover, according to Saracevic and Schlegelmilch (2021), identifying subjective norms is important to motivate sustainable practises, particularly regarding pro-environmental behaviour. In addition, Ramkissoon (2020) identifies that there is growing evidence of a link between health and wellbeing, and pro-environmental behaviour, and whilst the COVID-19 vaccine has proven 95% effective (Centers for Disease Control and Prevention [CDC], 2021), there are many individuals that remain unvaccinated.

#### Perceived Behavioural Control

Perceived behavioural control consists of two components: self-efficacy (the ease or difficulty of engaging in a specific behaviour), and controllability (the extent of individual performance) (Mai et al., 2021). It also indicates the importance of available resources and opportunities that contribute to a specific behaviour achievement (Ajzen, 1991). In addition, Ajzen (1991) explains that the idea of behavioural achievement depends on the intention through motivation, and ability through self-control. It has been recognised that previous studies have revealed there is a direct link between *attitudes* and *subjective norms* (Zhang et al., 2017), as social pressure that arises from other individuals’ beliefs or behaviours could facilitate or dictate the way a person behaves. Whilst Bandura (1977) recognised that behavioural control can produce positive outcomes, it is a complex task to foresee future decisions of people without understanding their behavioural attitudes and actions (Mai et al., 2021), particularly across the global tourism industry. Although Mai et al. (2021) identified there is a connection between subjective well-being and perceived behavioural control of international travellers, this is limited to predicting individuals’ intentions, not behaviour. As positive behavioural performance is motivated through the ability to remove individual psychological barriers (Hardin-Fanning and Ricks, 2016), a person’s behavioural control is also swayed by lower costs, time effectiveness, and adequate resources.

### Conceptual Model and Hypothesis Development

This study aims to identify whether individuals’ behavioural intentions demonstrate whether the perceived safety threat of COVID-19 affects their pro-environmental attitudes positively within the global travel industry. As the international travel industry begins to reopen, companies and organisations must inevitably adjust and amend policies and procedures, particularly regarding health, safety, and long-term environmental sustainability. This study uses certain constructs of the HBM and the TPB to gain a comprehensive insight into the pro-environmental behaviours of international travellers for leisure and recreational purposes.

As rendered by Rosenstock (1974), three factors that influence health-related action:

#### The Existing Concern of Making Health-Related Issues Relevant

As of 19 January 2022, 335,521,830 COVID-19 cases have been reported thus far, 5,574,726 deaths globally (Worldometer, n.d.). In addition, there are over 394,712 new cases and over 8,092 fatalities per day.

#### The Belief That Behaviours Create Vulnerability to a Specific Condition

The Centers for Disease Control and Prevention [CDC] (2021) declared that the COVID-19 vaccine rollout substantially reduced the risk of infection by 91% for those who have received full vaccination. Health and safety campaigns announced by the Centers for Disease Control and Prevention [CDC] (2021) and other governing officials have advised to be vaccinated, wear a face mask, and practise social distancing. It is noted, scientific studies have proven the effectiveness of these three precautionary measures, with 96.4% vaccine effectiveness (Pormohammad et al., 2021), 79% face mask effectiveness (Howard et al., 2021), and a substantial decrease in cases by adhering to social distancing, according to the results of a comprehensive study conducted by Matrajt and Leung (2020).

#### By Following Guidelines, Perceived Safety Threats Will Be Reduced, at a Justifiable Cost

An individual must believe that change of a specific kind will prove beneficial if it is set at an acceptable cost, whilst believing they can implement that change (Rosenstock, 1974). Several studies support the effectiveness of preventative measures, however, there is still scepticism amongst individuals globally, especially with a vaccine developed at “breakneck” speed (Abcarian, 2020), and a substantial number of online sources containing misleading and false information (Li et al., 2020). Based on the literature review, the authors proposed the following hypothesis.

**H1:** *Perceived safety threat has a positive impact on attitude.*

Rosenstock (1974) mentions that the outcome expectation is an individual’s chosen behaviour will determine a particular result. In addition, for a change in a specific behavioural pattern, a person must have an incentive to take relevant action, or possess a feeling of vulnerability, or be threatened, by their current behavioural patterns. Further, Rotter (1954) and Bandura (1977) suggest that an individual’s behavioural attitude is determined by expectancies and incentives regarding environmentally friendly practice. Expectations include environmental cues such as the belief about how events are connected, the consequence of one’s action, and self-efficacy. Incentives include an increase in health, physical appearance, approval of others, and economic gain. A positive outcome expectation will be measured against whether an individual’s attitude, perceived control, and the ability to influence others outweigh the risk susceptibility and risk severity whilst travelling during the COVID-19 pandemic, and thereafter, with current preventative measures in place. Consequently, the following hypothesis is proposed.

**H2:** *Outcome expectations has a positive impact on attitude.*

According to Ajzen (1991), identifying an individual’s chosen behaviour can be predicted with accuracy from that individual’s attitude, whilst capturing the motivational factors that initially influenced the behaviour. Campbell (1963) suggests that attitudes are residues of experience, with an individual’s unique contribution based on habit, rather than on reasoned action. Given the novel nature of the global outbreak, an individual’s knowledge and experience of COVID-19 may be limited. Therefore, results may produce a positive attitude regarding behavioural intention based on an individual’s reason, rather than based on habit, past knowledge, or experience. In addition, as outlined by Crano and Prislin (2008), we, as humans, favour some policies, whilst undermining others. As individuals and governmental bodies have demonstrated a strong emphasis on sustainable environmental practice and public health (Centers for Disease Control and Prevention [CDC], 2020), people were allowed to express their opinions regarding the use of travel and hospitality services that are pro-environmental. Based on hypotheses 1 and 2, people’s attitudes could affect international travel decisions, and choice of transportation during pandemics (Abdullah et al., 2020). Consequently, the authors postulate the following hypothesis.

**H3:** *Attitude has a positive impact on behavioural intention.*

Perceived pressure from society to conform, or not to conform to a specific behaviour is crucial to understand whether people are influenced by other individuals, family members, friends, loved ones, work or business partners (Ajzen, 1991). As previously mentioned, there is scepticism amongst adults regarding misinformation on social media sites, and mixed messages by government officials and scientists, particularly regarding vaccination and international travel (Abcarian, 2020). According to Ajzen and Fishbein (2000), as a general rule, the stronger a subjective norm, the more inclined an individual will perform the behaviour in question. Further, Vallerand et al. (1992) and Chang (1998), have demonstrated a significant existence between subjective norms and an individual’s behavioural intention (Al-Swidi et al., 2014). According to Yang et al. (2020), the subjective norm shapes people’s behaviours, as the fear of social rejection motivates individuals to act in accordance with referent groups. In a recent study conducted by Shin et al. (2022), results determined that individual’s travel decisions were significantly and positively impacted regarding subjective norms during the COVID-19 pandemic. Thus, this hypothesis will be tested to confirm whether individuals perform behaviours that fall in line with subjective norms, reflecting the social expectations that others have regarding an individual’s behavioural intention (Ajzen, 1991), particularly in the context of this research paper. Thus, on the basis of the above theoretical background, literature review, and empirical findings, the following hypothesis is proposed.

**H4:** *Subjective norm has a positive impact on behavioural intention.*

The idea of behavioural control is based on individual motivation, and is considered as an antecedent of behavioural intention (Ajzen, 1991). Further, Song et al. (2015) outline behaviour control as the perception of the ease or challenge in performing a behaviour. According to Mai et al. (2021), it is a difficult task to predict individuals’ future actions without understanding behavioural attitudes, particularly with the current situation of a global pandemic. In addition, perceived behavioural control has been associated with lower costs, time efficiency, and necessary resources (Hardin-Fanning and Ricks, 2016). Regarding the impact of COVID-19 and the awareness it has created around environmental practises (Lamers and Student, 2021), individual perception may have changed, or been influenced to utilise travel and hospitality services that are pro-environmental. In a study conducted by Aschwanden et al. (2021), findings suggest a strong connection between perceived behavioural control and preventative behaviours regarding the effect of COVID-19, such as hand washing, staying at home, using hand sanitiser and social distancing, especially with older adults. Therefore, this hypothesis aims to identify and validate the causal relationship between behavioural control and the positive impact on behavioural intention regarding COVID-19 and international travel. Consequently, the authors put forward the following hypothesis.

**H5:** *Perceived behavioural control has a positive impact on behavioural intention.*

**FIGURE 1:**
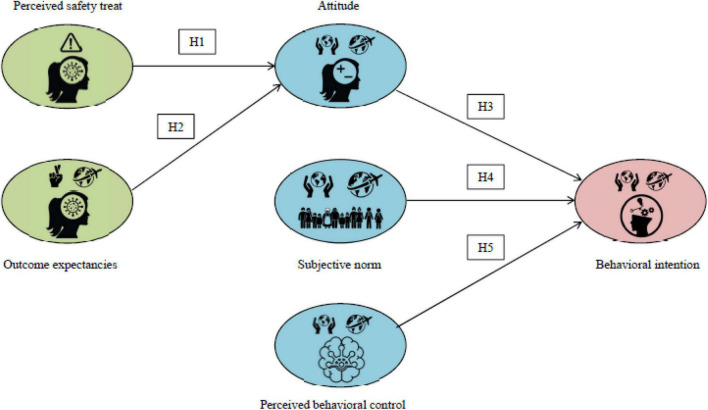
Integrative conceptual framework and proposed hypotheses.

In this context, the proposed hypotheses were based on individual knowledge and beliefs regarding risk susceptibility and severity of the COVID-19 virus. Further, this study sets out to test whether the preventative measures recommended by governing officials to reduce the perceived safety threat have had a positive effect on attitude, subjective norms, and perceived behavioural control toward international travel for leisure and recreational purposes. Below, Figure 1 shows the relationships between constructs and proposed hypotheses.

On reflection, the methodology section provides a clear and precise description of how the hypotheses were tested, and a rationale for the selected procedures critical for this specific topic. It is noted, the methodology was developed in line with the conceptual framework, which leads into the data analysis and results section.

## Methodology

As per the literature review, the HBM and the TPB models have been utilised with a confirmed validity across a vast number of socio-psychological studies, with relevant research topics conducted, tested, and proven by Huang et al. (2016) and Han et al. (2020). By applying a deductive approach, an explanation of causal relationships between the proposed hypotheses and their variables may present the ability to generalise findings to an extent and allow concepts to be measured in a quantitative capacity. This allows for a shorter time to complete the research and reduce the risk that the study will be influenced by pre-existing literature.

In this study quantitative approach was employed, as quantitative approach allows researcher to presents theories that are exemplified within specific hypotheses, which are then tested against relevant literature (Almalki, 2016). Lastly, gathered data through a quantitative measure will allow the application of a partial least-squares-structural equation modelling (PLS-SEM), which predicts variables based on the results of survey participants, and their observations (Hair et al., 2014), which, according to Usakli and Kucukergin (2018), has proved particularly beneficial in travel and hospitality research.

### Data Collection

A comprehensive online survey (please see Appendix 1) was constructed in English at SurveyMonkey^®^, an AI-powered platform, allowing a fast collection of data from online participation (SurveyMonkey, n.d.). SurveyMonkey was chosen for this study as it offers easy access, avoidance of input and data coding errors, and saves time and cost (Varela et al., 2016). Potential participants were invited to take part in the survey via social media platforms, including Facebook^®^, Instagram^®^, WhatsApp^®^, and LinkedIn^®^, using a mobile phone, tablet, laptop, or desktop. The main objective was to collect data from participants who have travelled internationally pre-COVID-19 pandemic (Pre-January 2020), travelled internationally during the COVID-19 pandemic (January 2020 – currently October 2021), and who plan to travel internationally in the future, post-COVID-19 pandemic. It is noted that targetting a broad, international sociodemographic range generates insight from individuals across many geographical locations. Thus, it is considered that participant results will differ across the nations included in the survey, particularly regarding risk susceptibility, risk severity, attitude, subjective norm, and perceived behavioural control.

### Measures

A cross-sectional, online, anonymous survey was developed with a comprehensive list of questions based on certain constructs of the HBM, the TPB model, and pro-environmental behaviour. The survey was a Likert scale (Sullivan and Artino, 2013), comprised of 21 adjective pairs, ranging from 1 = *extremely low*, to 7 = *extremely high*, and a bipolar scale including *bad-good*, *foolish-wise*, *unpleasant–pleasant*, and *harmful–beneficial*. Participants were asked to choose a number from 1 to 7 (including the bipolar scale options), that most closely described them, or their personal preference.

The scale items were adopted from the validated measurement items in the previous studies, and a Likert’s scale was used except for the demographic questions. Four items were adopted from Huang et al. (2016) to measure perceived safety threat. Four items were adopted from Han and Hyun (2019) and Severt and Tasci (2020) in order to measure outcome expectation. Lastly, remaining items to measure attitude toward pro-environmental behaviour, subjective norm, perceived behavioural control and pro-environmental behavioural intention were adopted from Han et al. (2020).

A rigorous method is an important part of assessing the legitimacy of a study, and to ensure trustworthy findings (Jordan and Troth, 2020). A procedural method was applied to avoid a common method bias, as neither the Harman test, nor the common factor method allows the elimination of the common method bias (Richardson et al., 2009; Conway and Lance, 2010). Therefore, the procedural strategies set by Jordan and Troth (2020) were used to minimise the common method bias were all participants were well-informed regarding the purpose of the research and how research results would be used. In addition, the survey used was not extensive, measurements were not overlapping, and the questions presented were transparent and without ambiguities (Jordan and Troth, 2020). This included the verbiage was constructed meticulously and adopted from various sources.

## Results

### Descriptive Statistics

A total of 331 respondents (125 males, 206 females) drawn from a variety of nations participated in the survey. The study attracted more women (62%) than men (38%). The age of participants was predominantly between 26 years old and 35 years old (44% of total respondents), followed by 36 years old to 45 years old (30% of total respondents). The majority of respondents held a university degree (38%), followed by those with a graduate degree (25%). Most participants were Caucasian/White (74%), the others were Asian, Hispanic, Black, and other (11, 7, 4, and 4% respectively). The monthly household income levels were well distributed, with most of the respondents earning between $55,000 to 69,999 (19%), followed by an income level of under $25,000, and $25,000 ∼ $39,999 (18% respectively). All participants were volunteers with no payment in return for completing the online survey. The descriptive statistics are provided below in Table 1.

**TABLE 1:** Demographic characteristics of the sample.

Demographic characteristics	Frequency	Percentage
**Gender**		
Male	125	38%
Female	206	62%
**Age group**		
25 years old and younger	18	6%
26–35	144	44%
36–45	98	30%
46–55	45	14%
56 or above	20	6%
**Income**		
Under $25,000	58	18%
25,000 ∼ $39,999	58	18%
40,000 ∼ $54,999	46	14%
55,000 ∼ $69,999	63	19%
70,000 ∼ $84,999	30	9%
85,000 ∼ $99,999	22	7%
100,000 or higher	54	16%
**Education**		
Less than high school degree	11	3%
High school degree	46	14%
2-year degree/community-college degree	66	20%
University degree	125	38%
Graduate degree	83	25%
**Ethnicity**		
Asian	36	11%
Black	12	4%
Hispanic	23	7%
Caucasian/White	246	74%
Other	14	4%
**Nationality**		
African countries	29	9%
Asian countries	35	11%
Central American countries	16	5%
North American countries	40	12%
South American countries	8	2%
European countries	199	60%
Other countries	4	1%

### Reliability and Validity Assessment

The reliability and validity were assessed in order to confirm the quality of the data and the consistency with the measurement items under the latent variables (Hair et al., 2019). To assess convergent validity, values relating to the average variance extracted (AVE) should be higher than 0.5 and the composite reliability (CR) of each construct should be above 0.6 (Fornell and Larcker, 1981). It is noted, AVE measures the number of variances captured by a construct, including the number of measurement errors (Afework et al., 2021). PL-SEM indices, including GFI, CFI, NFI, IFI, RFI, and TLI, should be greater than 0.90 to suggest a good model fit (Finch, 2019). Based on the results in Table 2 below, this indeed demonstrates a good model fit. The RMSEA, described by Xia and Yang (2018) as an absolute fit index that assesses how far the proposed hypothesised framework is from being a perfect model, should be less than 0.9 (Hu and Bentler, 1998). In addition, the SRMR, which is a robust method used to estimate the model parameters (Shi and Maydeu-Olivares, 2019), should also produce a value less than 0.9 (Hu and Bentler, 1998).

**TABLE 2:** Discriminant validity of conceptual model.

CMIN	DF	P	CMIN/DF	NFI	RFI	IFI	TLI	CFI	RMSEA
283.951	174	0	1.632	0.913	0.895	0.964	0.957	0.964	0.044

Below, Table 3 shows that all the values of AVE ranged from the satisfactory limit noted above (Fornell and Larcker, 1981). The internal consistency of measured items also indicated a good fit as CR, the measure of internal consistency in scale items (Fornell and Larcker, 1981; Netemeyer et al., 2003), also fulfilled the criteria of above 0.7 (Hu and Bentler, 1998). Both AVE and CR indicate good reliability of measured items (Bacon et al., 1995). In a CFA setting, the square root of AVE for each construct needs to be greater than its squared inter-construct correlation estimates to achieve discriminant validity (Hair et al., 2009), defined as a set of empirical criteria that can be assessed from multitrait-multimethod matrices (Rönkkö and Cho, 2020). The results show that the square root of AVE ranged from 0.506 to 0.786, exceeding the cut-off value of 0.5 (Fornell and Larcker, 1981). In addition, the factor loading of each item is above the recommended threshold of 0.5 (Zhu and Deng, 2020). The results indicate there are no serious occurrences of discriminant validity in this study. Table 3 also illustrates and confirms that all AVE and CR values meet the threshold criteria.

**TABLE 3:** Correlations among latent constructs.

Factors	CR	AVE	Perceived safety	Outcome expectation	Attitude	Subjective norm	Perceived behavioural control	Behavioural intention
Perceived safety	0.752	0.539	**0.663**					
Outcome expectation	0.644	0.506	–0.18	**0.563**				
Attitude	0.859	0.606	–0.011	0.13	**0.799**			
Subjective norm	0.915	0.782	0.06	0.324	0.449	**0.885**		
Perceived behavioural control	0.797	0.573	–0.047	0.297	0.21	0.36	**0.757**	
Behavioural intention	0.873	0.698	0.016	0.335	0.336	0.66	0.623	**0.835**

Furthermore, as PLS-SEM suffers from criticisms of flexibility compared with CB-SEM a normality test was performed and Table 4 outlines normality test of the variables.

**TABLE 4:** Assessment of normality.

Variable	Min	Max	Skew	c.r.	Kurtosis	c.r.
Outcome expectation	–2.629	1.143	–0.835	–6.202	1.310	4.865
Perceived safety	–1.738	2.140	0.463	3.441	–0.248	–0.922
Perceived behavioural control	–3.838	1.265	–0.884	–6.568	1.576	5.853
Subjective norm	–3.938	1.903	–0.571	–4.238	0.323	1.201
Attitude	–3.974	0.611	–2.202	–16.354	5.341	19.834
Behavioural intention	–4.255	1.594	–0.722	–5.361	1.318	4.893

The skewness of all the variables was between –1 and 1 except the value for attitude. The attitude was negative skewed and its value is –2.202. Likewise kurtosis of all the variables is acceptable except for the attitude. However, as PLS-SEM is not effected by the distribution of the data, further analysis was done with respect to the proposed hypotheses.

### Structural Model

**FIGURE 2:**
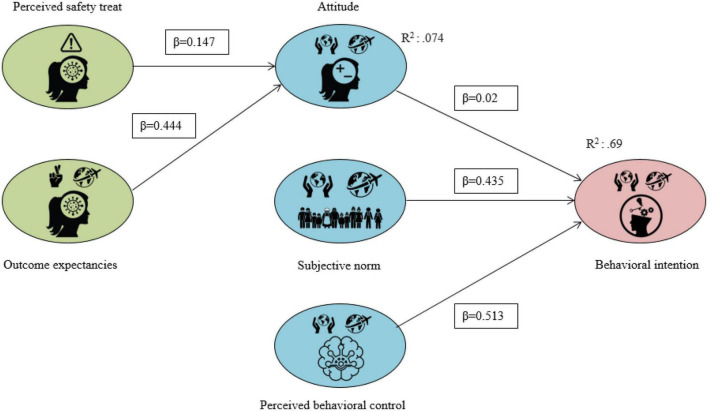
Result of the structural conceptual framework model.

The maximum-likelihood-estimation (MLE), which is a preferred method of parameter estimation in statistics is an indispensable tool for statistical modelling techniques (Myung, 2003). This was used to examine the relationships among perceived safety threat, outcome, attitude, perceived control, subjective norms, and behavioural intention (see Table 5). The results confirm that the full structural model (H1–H5) is a good model fit (CMIN/DF = 2.044; NFI = 0.895; RFI = 0.864; IFI = 0.944; TLI = 0.926; CFI = 0.943; RMSEA = 0.056; AND SRMR = 0.036), as per Table 6 below. As both the RMSEA and the SRMR produced values of 0.056 and 0.036 respectively (see Table 6), this suggests the model is a good fit, as the results are below the 0.9 thresholds (Xia and Yang, 2018; Shi and Maydeu-Olivares, 2019). Moreover, the results in Table 2 (χ2 = 283.951, df = 174, RMSEA = 0.044, CFI = 0.964, TLI = 0.957, IFI = 0.964, NFI = 0.913 yield a good model fit). Thus, the results of the measurement model ensured that the study has achieved construct reliability and validity. Figure 2 shows the results of the structural conceptual framework model.

**TABLE 5:** Hypothesis testing – MLE (maximum likelihood estimation).

Hypothesis				Estimate	*SE*	*P*-value
H1	Attitude	<—	Safety	0.147	0.104	*
H2	Attitude	<—	Outcome	0.444	0.101	***
H3	Intention	<—	Attitude	0.02	0.046	*
H4	Intention	<—	Norm	0.435	0.044	***
H5	Intention	<—	Control	0.513	0.064	***

**TABLE 6:** Discriminant validity of final model.

CMIN/DF	NFI	RFI	IFI	TLI	CFI	RMSEA	SRMR
2.044	0.895	0.864	0.944	0.926	0.943	0.056	0.036

The proposed conceptual framework was deemed adequate for the relationship between the proposed hypotheses and study variables. The proposed conceptual model was deemed adequate for the total variance in attitude (7.4%) and behavioural intentions (69.4%), which were all, as per Aneshensel (2013), above the acceptable levels for studies in social sciences. Reflecting on Table 5 and Figure 2, the perceived safety threat has a significant effect on attitude (β = 0.147, *p* < 0.001) and attitude has a significant effect on perceived behavioural intentions (β = 0.02, *p* < 0.001). Therefore, Hypotheses 1 and 3 are supported. Further, the hypothesised relationship of outcome expectancies on attitude was positively affected (β = 0.444, *p* < 0.001), supporting Hypothesis 2. Moreover, the positive link between the subjective norm and behavioural intention is statistically significant (β = 0.435, *p* < 0.001), supporting Hypothesis 4. Lastly, perceived control was positively influenced by perceived behavioural intentions (β = 0.513, *p* < 0.001), confirming Hypothesis 5.

The results have confirmed and supported the hypotheses, including the relationships with latent constructs. Therefore, Table 5 outlines and exhibits the accepted conceptual framework (H1–H5), producing a significant, and positive outcome. To delve further into the meaning, relevance and importance of the results, the discussion section expands on the relationship between the literature review and proposed research questions.

## Discussion

The findings relating to the effect of COVID-19 on pro-environmental behaviour of individuals travelling internationally for leisure and recreational purposes, offer a significant contribution to current literature, including the five key components consisting of perceived risk susceptibility, perceived risk severity, attitude, subjective norm, and perceived behavioural control. From the findings, it appears that the hypotheses demonstrate a viable, and causal relationship, comprising five distinct variables based on existing literature.

The perceived safety threat (risk susceptibility) significantly impacted attitude, which confirms the three theoretical factors outlined by Rosenstock (1974) that influence individuals taking health-related action. With that being said, the first factor relates to the existence of sufficient concern regarding the effect of COVID-19, and the heightened perception of risk susceptibility toward health-related issues, particularly with international travel. Secondly, the vulnerability of one’s behaviour is amplified, resulting in a reluctance to travel internationally for leisure and recreational purposes whilst COVID-19 is still prevalent. Lastly, individuals are more likely to follow the recommended guidelines set by governmental officials, such as wearing a face mask, socially distancing from others, staying at home, receiving the COVID-19 vaccination, washing hands and self-isolating if there are developing symptoms.

Although it is suggested that risk susceptibility affects individuals’ attitudes positively, according to Zhu and Deng (2020), risk susceptibility is merely the starting point for a judgement of crisis impact on the tourism market. With this considered, the outcome of an individual’s risk susceptibility suggests that the COVID-19 cycle is still in the early stages of the crisis. Further, an individual’s attitude is subject to significant influence from perceived safety threats (Zhu and Deng, 2020), with some studies suggesting that an individual’s perceived susceptibility is of greater interest than the perceived value of travelling. In addition, individuals may only decide to travel if guidelines set by governmental officials are being followed, particularly regarding individuals receiving the COVID-19 vaccine. Inevitably, according to studies conducted by Polack et al. (2020), receiving the vaccination would lessen the risks of contracting COVID-19 by 95%. Moreover, the length of time required to reduce the level of scepticism amongst individuals, particularly with the rapid development of the COVID-19 virus vaccine, may highlight implications for organisations in the global travel industry. Lastly, tourism risk is often associated with a service or product that has gone beyond control after the process of travel has begun (Cui et al., 2016). Indeed, this is particularly true for the spread of the COVID-19 virus. Therefore, individuals may only begin to travel once nationals of the country travelled to, or individual travellers have received the COVID-19 vaccine, particularly regarding travel to well-known tourism hot spots.

According to Rosenstock (1974), an individual must benefit from changing their current behaviour patterns to counteract the feeling of vulnerability or threat regarding a particular situation. The testing of this hypothesis supports the positive reflection of individuals’ attitudes toward risk susceptibility, as the outcome expectation is measured against whether an individual’s attitude, the influence of others, or perceived behavioural control outweigh the risk susceptibility of contracting the COVID-19 virus. It is noted, however, expectations are the variations of beliefs (Hsu et al., 2009), such as distinctive demographic characteristics and behavioural patterns of identified market clusters that relate to an individual’s action, and whether a decision produces a favourable, or unfavourable outcome. Consequently, one can motivate, or influence another, and subjectively manipulate the outcome expectation. In addition, demand for tourism significantly decreased because of the COVID-19 pandemic (Kim et al., 2021), with tourists expecting a less crowded “travel-to” destination.

Although results confirm positive behavioural changes to offset the feeling of vulnerability and threat, the question remains whether companies and organisations are responding to behavioural studies outlining consumer concerns, and expectations. However, according to Bielecki et al. (2020), there has been a fundamental challenge regarding traveller communication concerning air travel restrictions, including passenger number limitations, testing requirements and associated costs, quarantine measures, and enforcing governmental rules of wearing masks, washing hands, and socially distancing. The confusion with mixed messages from officials announcing reduced preventative measures, versus heightened measures with travel industry companies, may deter individuals further. Consequently, this causes increased frustration for individuals who wish to travel internationally (Escandón et al., 2021).

The outcome of this hypothesis has supported the previously mentioned study conducted by Perrotta et al. (2021), suggesting individuals’ attitudes had changed significantly, with people adopting the use of facemasks, hand washing and social distancing before it was declared mandatory by governmental officials. As explained by Han et al. (2020), in conjunction with individuals’ attitude toward risk susceptibility, it is identified that attitude has a similar outcome regarding individual behavioural intention: the decrease of anxiety and risk attitude increases travel intention.

As a result of individuals adhering to preventative measures set by governmental officials and global leaders to stay at home and limit travel, the global travel economy has been devastated. A further point to consider is that the findings from testing this hypothesis relate to individuals travelling internationally for leisure and recreational purposes in general, and don’t specifically relate to individuals travelling to visit friends or family members, or people travelling for work or business purposes. In a study conducted by Das et al. (2021), approximately 85.7 million immigrants who currently reside in the United States, are unable to return to their native land to visit and care for family members who have been affected by the COVID-19 pandemic. Although the study may have tested positively regarding individuals travelling internationally in such circumstances, this presents an opportunity for continued research relating to COVID-19 and individuals’ pro-environmental behaviour from a personal and business perspective, rather than solely for leisure and recreational purposes.

The survey presented three questions for the participant to identify whether the level of influence from others, those whose opinion matters, and those who are important in their lives, holds value regarding the decisions made to use travel and hospitality services that are pro-environmentally friendly. Indeed, the findings from the sample suggest that individuals are more inclined to use pro-environmental services in the future. According to Sujood and Bano (2021), there is substantial societal pressure to abide by preventative behaviour, and if family members or peers have a biassed attitude regarding preventative measures, the probability of an individual following the same behaviour would be likely high.

However, certain factors must be taken into consideration, such as the sociodemographic range of survey participants. Firstly, the Centers for Disease Control and Prevention [CDC] (2020) has determined that COVID-19 related deaths are predominantly among individuals aged 45 + years old. This study consisted of 65 individuals aged 46 and older (20% of all survey participants), suggesting that risk susceptibility and risk severity highlighted by governmental officials may pose a greater influence rather than individuals whose opinion matters most to the participant. Moreover, according to Hamer et al. (2018), ethnic minority group classification is vague, with no agreement on the definition of “ethnicity” by researchers across various disciplines in social science studies. Thus, there is ambiguity in defining “ethnic minorities” amongst researchers and how laypeople define the term. The Centers for Disease Control and Prevention [CDC] (2020) suggest ethnic minorities are amongst those deemed most at risk, including age and underlying health concerns. This study was skewed, as 74% of participants were of White/Caucasian ethnicity, and 26% were of Asian, Hispanic, and Black ethnicity. It is noted, survey participants may have answered the questionnaire with influence from individuals whose opinions are valued. However, the questions presented in the survey focus on the subjective norm, rather than identifying the origin of where information was obtained, such as credible sources outlining facts and statistics published by scientists and governing officials, or, media sources that portray false, or misleading information.

The positive outcome of individuals’ perceived behavioural control suggests that the necessary change of behaviour is relatively simple to achieve if the individual is (1) self-motivated, and, (2) has the necessary capability to do so (Ajzen, 1991). In addition, the findings suggest that perceived behavioural control is linked with an individual’s attitude and the subjective norm (Tarkiainen and Sundqvist, 2005; Mai et al., 2021), suggesting individuals are influenced by others, and information received from other sources (Zhang et al., 2017). However, considering the divide between individuals influenced by personal and close relationships (direct), versus the influence caused by media announcements (indirect), a fundamental challenge remains for the global tourism industry regarding consumers’ behavioural intention. This is significant, particularly with the number of mixed messages delivered by various accessible sources, government officials, scientific experts, and worldwide leaders (Zampetakis and Melas, 2021).

The findings from the survey suggest individuals were in favour of choosing pro-environmental travel and hospitality services, but, this isn’t necessarily a true reflection of the final purchasing intention. In a survey conducted by White et al. (2019), 65% of consumers said they had good intentions to purchase “green,” environmentally friendly, and sustainable services, but, only 26% did. Further, according to O’Connor and Assaker (2021), the relationship between global pandemics and travel/tourism has predominantly focussed on the financial and economic angle of general travel and tourist destinations. Consequently, the long-term, non-economic implications, such as the mental health and well-being of individuals (Knolle et al., 2021), and psychological and social effects of individuals (Calbi et al., 2021), are unaccounted for, particularly regarding the change in international travellers’ behaviour (O’Connor and Assaker, 2021). Thus, individuals may have good intentions and are motivated to purchase sustainable, pro-environmental travel services, however, may not possess the necessary resources required to purchase environmentally sustainable options.

This study has focussed on the gap in current literature regarding the challenges outlined in the research questions. In addition, the objectives have been addressed, underpinning the theoretical and practical implications of the effect of COVID-19 is having on pro-environmental behaviours of leisure-orientated international travellers. Findings suggest there may still be many hurdles to recovering a collapsed global travel industry. Moreover, companies and organisations must promptly adjust policies regarding environmental practises, and strategically manoeuvre business objectives, given the impact of COVID-19, and its influence on the pro-environmental behaviour of international travellers.

### Practical Implications

Based on Zhu and Deng (2020), there is a link between risk susceptibility and the judgement of crisis in the global travel industry. With that considered, industry practitioners and researchers must conduct frequent investigations to identify how to reduce an individual’s susceptibility to the risks of international travel. This will provide significantly important updates to companies and organisations within the travel sector, allowing a shift in management and marketing strategies, including necessary adjustments to environmental policies and procedures. In addition, the frequent review of risk susceptibility would prepare the travel industry for the moment individuals decide to resume international travel based on a reduction in the existence of threats regarding the COVID-19 pandemic. Further, concerns about the unvaccinated, and those who are reluctant to follow governmental guidelines may heighten individuals’ level of risk susceptibility and how they perceive the threat of contracting COVID-19. Therefore, it is necessary for governmental officials to regularly update companies and organisations to allow them to communicate and publish positive trends regarding vaccinations provided to home nationals. This holds the potential to reduce the threat and risk severity of the COVID-19 virus and entice individuals to resume international travel, particularly to hot spot tourist destinations.

Regarding mixed messages, government officials and global travel industry leaders must communicate the same message, given there is substantial tension between government officials whose primary objective is to protect the health and well-being of individuals, whilst global travel companies and organisations aim to generate profits (Ratten, 2020). This will increase peace of mind among those keen to travel in specific periods throughout the year. It may be in the best interest of industry companies and organisations to offer a full refund for any COVID-19 related circumstance, to rebuild long-term relationships with consumers. Global travel companies and organisations must further investigate alternative reasons for international travel rather than solely leisure and recreational purposes, such as visiting friends and family, and business professionals travelling for work. This would provide further insight into the attitudes of those for whom travel is a necessity, rather than a luxury, and support the travel industry to target the various needs and travel intentions of individuals.

Moreover, it is suggested age and ethnic demographics should be investigated further, including direct (those who provide valued opinions), or indirect (the media, scientists, and government officials) subjective norms to better understand the influencing factors associated with behavioural intentions. Further, it is necessary to understand behavioural intentions that relate to cost-effective pro-environmental services, as this, according to many studies, suggests individuals have good intentions to choose environmentally sustainable international travel options, but that the decision is swayed when factoring in an individual’s financial resources. Therefore, it may be necessary to further investigate the extent of resources individuals are willing to exchange before the perceived value of pro-environmental travel becomes a financially viable option. This also supports companies and organisations to provide alternative cost-effective services that are sustainable or add value to entice individuals to select specific services. In addition, future studies would benefit from identifying and specifying alternative sources that influence subjective norms, including the influencing factors from both people whose opinions matter the most, and external credible, and non-credible sources. As recent studies have also found over a quarter of online information is either false or misleading (Li et al., 2020), it may be necessary to identify which platform the participant used to gather the information that influenced the individual’s behavioural intention.

### Theoretical Implications

Regarding the conceptual framework, it is noted that this study predominantly focuses on two of the constructs of the Health Belief Model (Rosenstock, 1974), namely risk susceptibility and risk severity. Given the novel nature of the COVID-19 pandemic, an initial investigation of the constructs was necessary to identify the behavioural intentions of international travellers at a time of significant importance, such as the reopening of the global travel industry. Before the vaccine rollout, a survey conducted by Wong et al. (2020), consisting of 1,159 participants, found that an individual’s perceived benefit from receiving a COVID-19 vaccine dose would be a lower chance of contracting the COVID-19 infection, making them less worried about virus severity and susceptibility. As there have been remarkable vaccination developments since this study was conducted, the rapid development and administration of the COVID-19 vaccine has caused a fundamental shift in behavioural attitude, as individuals have become increasingly sceptical, particularly with mixed messages, and misleading information (Li et al., 2020).

Comparing the results of this study versus the study results outlined by Wong et al. (2020), it is evident there is a notable change in an individual’s behavioural intention, especially regarding the perceived barriers which are influenced by the attitude, the subjective norm, and the perceived behavioural control relating to individuals and the COVID-19 vaccination. Consequently, this adversely affects the two additional constructs, namely cue to action, an individual’s ability to identify risk factors of the COVID-19 virus, and self-efficacy, the belief that a particular behaviour can influence a desirable outcome. As with the perceived benefits, this may result in a continuous change of an individual’s behavioural intention (Glanz et al., 2015). With risk susceptibility and risk severity identified, further research into the additional four constructs previously mentioned would provide a valuable extension of an individual’s behavioural intention to support companies and organisations in the international travel sector.

### Limitations and Future Research

This study didn’t come without its limitations. Regarding the quantitative research method, future studies should consider an adjustment to the Likert scale, offering a simple “yes” or “no” answer to collect a more definitive response. Further, the survey included more than three-quarters of individuals from a White/Caucasian background, with the sample predominantly aged between 26 years old to 35 years old. Future research would therefore benefit from incorporating more individuals from different nations, including a wider range of age groups. This would provide greater insight and more depth to the study, particularly with participants from societies who may possess alternate cultural perspectives. Moreover, this study contains a broad sociodemographic range with a general overview of travel methods within the global travel industry. Further research would benefit from identifying each sociodemographic category, and each type of travel used, which would offer companies and organisations within the global travel industry a specific strategy to support sustainability, as well as economic growth. In addition, this study includes respondents who have travelled outbound internationally before and during the outbreak of the COVID-19 pandemic with a variety of different and limited travel experiences. This may affect participants responses to the survey, suggesting there may be a need to identify the frequency of individuals’ level of international travel, and categorise respondents accordingly for further insight into traveller’s behavioural intentions. Lastly, this study consisted of a cross-sectional time horizon, focussing on a specific point in time regarding the study topic. Ongoing studies should consider both a cross-sectional time horizon method, and a longitudinal research method, to review and analyse data over a longer, more sustained period.

## Conclusion

The impact of the COVID-19 pandemic across the globe is still very uncertain. Some of the most profound effects so far include local, national, and international travel restrictions, suggesting the COVID-19 pandemic is still at the starting point for a judgement of crisis. In addition, although there has been substantial development of the COVID-19 vaccine, there are still many individuals worldwide that have yet to receive the vaccination, particularly home nationals of countries listed as the most visited destinations in 2019. Moreover, there are fundamental challenges regarding rules and regulations with international travel, such as COVID-19 quarantine measures, testing requirements and associated costs, confusing international travellers. Moreover, non-economic implications, such as mental stability, and the psychological and social effects of individuals, may affect an individual’s decision to travel, or choose to purchase pro-environmental services and products.

This research has set a foundation for the effect of COVID-19 on the pro-environmental behaviour of international travellers and will form the basis for further investigation into the previously mentioned practical implications. Given the novel nature of the COVID-19 pandemic and the unpredictability of human behaviour, the Health Belief Model and TPB frameworks must be utilised to test the behavioural intention of individuals who choose to travel internationally, for any reason. As the travel industry slowly reopens, strong communication is essential between governmental officials and consumers planning to travel, to instil trust, whilst reducing the fear and threat of the COVID-19 symptoms. Lastly, companies and organizations need to balance a fair cost regarding pro-environmental travel options while allowing access to those who are greatly influenced by the financial commitment to utilising such services.

## Data Availability Statement

The raw data supporting the conclusions of this article will be made available by the authors, without undue reservation.

## Author Contributions

All authors listed have made a substantial, direct, and intellectual contribution to the work, and approved it for publication.

## Conflict of Interest

The authors declare that the research was conducted in the absence of any commercial or financial relationships that could be construed as a potential conflict of interest.

## Publisher’s Note

All claims expressed in this article are solely those of the authors and do not necessarily represent those of their affiliated organizations, or those of the publisher, the editors and the reviewers. Any product that may be evaluated in this article, or claim that may be made by its manufacturer, is not guaranteed or endorsed by the publisher.

## APPENDIX

**Appendix 1:** Constructs and measurement items.

Constructs and Items
**Perceived safety threat (Huang et al., 2016)**
1. My chance of getting contracted by COVID-19 while using travel and hospitality services for leisure [Extremely low (1) – (7) Extremely high]
2. Because of my physical health, I am more likely to be infected by COVID-19 if I use travel and hospitality services for leisure [Extremely low (1) – (7) Extremely high]
3. The thought of suffering COVID-19 scares me [Extremely low (1) – (7) Extremely high]
4. My financial security would be endangered if I had COVID-19 [Extremely low (1) – (7) Extremely high]
**Outcome expectation (Han and Hyun, 2019; Severt and Tasci, 2020)**
1. I use travel and hospitality services to interact with my friends and family (Extremely low (1) – (7) Extremely high)
2. Travelling for leisure is truly a joy (Extremely low (1) – (7) Extremely high)
3. Compared to the price I paid, I think I will receive good value while using travel and hospitality services (Extremely low (1) – (7) Extremely high)
4. Travelling for leisure will compensate for what I miss in my daily life during the COVID-19 pandemic period (Extremely low (1) – (7) Extremely high)
**Attitude toward pro-environmental behaviour (Han et al., 2020)**
1.Using travel and hospitality services that are pro-environmental is:
2. Bad (1) – Good (7)
Foolish (1) – Wise (7)
3. Unpleasant (1) – Pleasant (7)
4. Harmful (1) – Beneficial (7)
**Subjective Norm (Han et al., 2020)**
1. People who influence my behaviour would think that I should use travel and hospitality services that are pro-environmental [Extremely low (1) – (7) Extremely high]
2. People who are important to me would think that I should use travel and hospitality services that are pro-environmental [Extremely low (1) – (7) Extremely high]
3. People whose opinions that I value would prefer that I use travel and hospitality services that are pro-environmental [Extremely low (1) – (7) Extremely high]
**Perceived behavioural control (Han et al., 2020)**
1. Whether I use travel and hospitality services that are pro-environmental is entirely up to me [Extremely low (1) – (7) Extremely high]
2. I am confident that I can use travel and hospitality services that are pro-environmental [Extremely low (1) – (7) Extremely high]
3. I have sufficient resources, time, and opportunities to use travel and hospitality services that are pro-environmental [Extremely low (1) – (7) Extremely high]
**Pro-environmental behavioural intention (Han et al., 2020)**
1. I plan to use travel and hospitality services that are pro-environmental [Extremely low (1) – (7) Extremely high]
2. I will exert effort to use travel and hospitality services that are pro-environmental [Extremely low (1) – (7) Extremely high]
3. I am willing to use travel and hospitality services that are pro-environmental [Extremely low (1) – (7) Extremely high]
